# Prognosis Analysis and Clinical Features of Orbital Cavernous Venous Malformations With Refractory Insidious Onset

**DOI:** 10.3389/fonc.2021.745479

**Published:** 2022-02-01

**Authors:** Peng Yang, Yong Li, Hao-Cheng Liu, E. Qiu, Jia-Liang Zhang, Jian Ren, Li-Bin Jiang, Hong-Gang Liu, Jun Kang

**Affiliations:** ^1^ Department of Neurosurgery, Beijing Tongren Hospital, Capital Medical University, Beijing, China; ^2^ Department of Neurosurgery, Xuanwu Hospital, Capital Medical University, Beijing, China; ^3^ Department of Ophthalmology, Beijing Tongren Hospital, Capital Medical University, Beijing, China; ^4^ Department of Pathology, Beijing Tongren Hospital, Capital Medical University, Beijing, China

**Keywords:** orbital cavernous haemangioma, optic nerve sheath, orbital apex, the common tendon ring, orbital cavernous venous malformations

## Abstract

**Objective:**

The present study aims to analyse the clinical presentation, treatment and prognosis of a group of patients with orbital cavernous venous malformation (OCVM) with an insidious onset.

**Method:**

The clinical data of 35 patients with OCVM treated at our centre between 2003 and 2020 were retrospectively analysed. The OCVMs were classified as one of six types (I–VI) according to the orbital position of the tumour. The clinical characteristics, treatment methods and follow-up results were recorded.

**Results:**

A total of 35 patients with OCVM under the optic nerve sheath in the orbital apex area or the common tendon ring (Types I and II) were included in the present study. In 20 cases (57.1%), patients were misdiagnosed with optic neuritis, and in 20 cases (57.1%), the tumour was not identified based on imaging. The presentation was acute or subacute in 23 cases (65.7%). All patients underwent surgery: transnasal surgery in 22 cases (62.9%) and craniotomy in 13 cases (37.1%). A total of 9 patients (25.7%) experienced postoperative complications, and 17 patients (48.6%) experienced vision improvement. The average patient age at first diagnosis was 43.3 ± 10.3 years, and the median follow-up period was 64.5 months. Overall, 14 patients (40%) experienced postoperative complications: postoperative blindness in 6 cases, postoperative vision loss in 8 cases and orbital apex syndrome in 7 cases.

**Conclusion:**

Patients with Type I and Type II OCVMs are the most complex cases. They have an insidious onset and are associated with a high rate of misdiagnosis and missed diagnosis. Acute and subacute decreases in visual acuity are mainly caused by OCVM haemorrhage. The difficulty of surgical treatment and the poor prognosis of postoperative vision are characteristics of this tumour. Transnasal surgery and craniotomy can be used to remove OCVMs located in the common tendon ring or optic canal as well as those involving the intracranial area through the supraorbital fissure. Meanwhile, the orbital approach (orbitotomy) has proven to be an effective method of treating OCVMs not involving the deep orbital apex and intracranial area.

## Introduction

Orbital cavernous venous malformation (OCVM) is a common benign tumour of the orbit occurring in adults and accounting for 13–22% of all orbital tumours (1, 2). An OCVM is a venous malformation, also referred to as a cavernous vascular malformation (3, 4). It is the most common type of orbital vascular malformation (5) and is sometimes present in combination with other vascular malformations (6).

Once found, orbital cavernous haemangioma (OCH) is usually surgically resected. The tumour has a complete capsule, and a tumour located in or outside the orbital muscle cone has no evident adhesion to the orbital contents. Its clinical manifestations are primarily protrusive eyes, and it can generally be completely resected with few surgical complications.

However, there is an OCH type that does not present as protrusive eyes; it is characterized by a hidden onset, difficult detection and treatment, rapid disease progression and poor clinical prognosis.

An OCVM can occur in any part of the orbit; those situated in the deepest part of the orbit, namely the common tendon ring and the optic canal of the orbital apex, are the most difficult to treat. Deep OCVMs generally occur at the common tendon ring in the extraocular muscle cone or the optic nerve sheath; the tumours are deep and adjacent to vital structures, such as the optic nerve, extraocular muscles and intra-orbital blood vessels. Consequently, surgery in this area is challenging and risky.

In addition, tumours located in the deep part of the orbit are usually small in size, and therefore do not exert a space-occupying effect. As a result, they are not easy to find in the early stages, and the rate of clinical misdiagnosis and missed diagnosis is therefore extremely high.

Bleeding of the tumour can result in an acute decline in vision, leading to late diagnosis in the clinical course (7). The overall five-year intracranial haemorrhage risk from all types of CMs in the brain is approximately 15.8%, and the annual incidence of bleeding from spinal cord CMs is approximately 2.1%. However, the incidence of bleeding from OCVMs has not been adequately reported in relevant literature.

OCVMs have a potential risk of rupture and haemorrhage, which can cause irreversible blindness. Therefore, the authors of this study believe that early diagnosis and strict follow-up should be conducted for OCVMs located in the deep orbital apex or near the optic canal (Types I and II). Because these kinds of OCVMs are small and deep, the operation is difficult, and the complication rate is high. The surgical indications are questionable (8); however, we should be highly alert during the clinical follow-up regarding sudden visual loss caused by OCVM rupture and haemorrhage. The authors of the present study believe that when OCVMs (such as Types I and II) rupture and haemorrhage or the patient has a sudden vision loss, positioning and qualitative diagnosis before surgery are crucial.

In the present study, 35 cases of OCVM with imaging appearance, histopathology and surgical approaches were researched; the clinical features and prognosis were then compared according to tumour position type.

## Methods

The clinical data of 35 patients with OCVM treated at our centre between January 2003 and October 2020 were collected and retrospectively analysed. The study met the requirements of the World Medical Association’s Declaration of Helsinki and was approved by the ethics committee of our centre. The patients or their family members provided informed consent.

The clinical characteristics of this series of patients with insidious onset OCVM were analysed. Each OCVM was then classified as one of six types (I–VI) based on the orbital position of the tumour and its positional relationship to the bony orbit, eyeball, optic nerve canal within the orbit and communication area inside and outside the orbit.

The classification criteria were mainly based on the characteristics of all our retrospective clinical cases. Six main types of OCVM were broadly outlined based on the tumours’ different anatomical locations in the cranio-orbital region (location within or outside the musculo-vertebral region, relationship with the optic nerve, involvement of extra-orbital or even intracranial structures, etc.), and hand-drawn diagrams were provided.

Moreover, it was also on the basis of the prognosis of the different clinical disease subtypes brought about by these different anatomical OCVM features that a part of the quite distinctive clinical case features was synthesized.

All patients were treated surgically with either a transnasal endoscopic approach or a transfronto-orbital approach. The appropriate surgical approach was conducted according to the different anatomical tumour locations in the orbit; an effort was then made to adopt the most suitable method for tumour removal, i.e., avoiding damage to the optic nerve and extraocular muscles was required while removing the tumour.

Transnasal endoscopic approach: With the combined neuronavigation and endoscopic operation, a single nostril approach to the lateral middle turbinate was conducted; after incising the septal vesicle, the pterygoid sinus was entered and the optic canal exposed.

According to the different anatomical locations of the tumour, the bone of the orbital wall in the orbital apical region and the bone of the wall in the optic canal were removed by pneumatic drilling. The superior, medial and inferior walls of the optic canal were also removed by 270° grinding. The tumour was finally exposed and removed *via* orbital periosteum incision.

Transfronto-orbital approach: A frontotemporal arcuate incision was made, the frontotemporal bone flap on one side and the brow arch bone flap on the other side were removed by milling, and the supraorbital wall and the optic nerve canal wall were abraded. The bony structure of the supraorbital fissure was then fully opened, and the tissue structure inside the supraorbital fissure was completely exposed. Next, the intra-orbital tumour was exposed under the microscope, with the full protection of the optic nerve, the extraocular muscle and the neuromuscular structure inside the general tendon ring. Finally, the tumour was completely removed.

Transnasal surgery was used to remove tumours located on the medial or inner-lower side of the optic nerve at the orbital apex. A craniotomy microscope is often used to cut the total tendon ring and remove tumours located on the upper side of the optic nerve at the orbital apex. When a tumour is located on the temporal side of the optic nerve at the orbital apex, a transorbital approach or a craniotomy can be used to remove the tumour with the aid of a microscope. The general principle is to minimize the surgical approach of the most important orbital structure in order to remove the tumour.

The researchers in the present study recorded the patient clinical characteristics, surgical approaches, computed tomography (CT) and magnetic resonance imaging (MRI) results, pathology specimens and follow-up outcomes.

## Results

### Clinical Outcomes

The study included 35 patients with OCVM; of these, 10 (28.6%) were male and 25 (71.4%) were female. The average age at first diagnosis was 43.3 ± 10.3 years, and the median follow-up period was 64.5 months. In 20 cases (57.1%), the patients were misdiagnosed with optic neuritis, and in 20 cases (57.1%), the tumour was not identified based on imaging. The presentation was acute or subacute in 23 cases (65.7%). All patients underwent surgery: transnasal surgery in 22 cases (62.9%) and craniotomy in 13 cases (37.1%). Postoperative complications were present in 9 cases (25.7%), and vision improvement was present in 17 cases (48.6%).

### Imaging Characteristics

All OCVMs were Type I or Type II; a total of 9 were located under the optic nerve sheath in the orbital apex area, and 26 were found in the common tendon ring region. On T1-weighted images, all OCVMs appeared to be homogeneous and isointense. On the T2-weighted images, the tumour appeared hyperintense, homogeneous and heterogeneous; hyperintensity was characteristic. The most common enhancement mode was multiple patchy starting points distributed throughout the tumour, finally becoming homogeneous or heterogeneous in the later stage.

In 20 patients, no tumour was identified in the brain MRI, and the diagnosis was confirmed using either optic nerve or orbital MRI.

All patients had an orbital or optic canal CT confirming the absence of bone hyperplasia, absorption, or destruction caused by the OCVM and showing no obvious OCVM calcification.

### Histopathology and Immunohistochemistry Characteristics

All 35 patients included in the study underwent surgical tumour removal, and all samples obtained during the operation were confirmed as originating from an OCVM. Surgical specimens were collected for histological and immunohistochemical analyses. Immunohistochemical studies for CD34, CD31, CD99, SMA, Vimentin, P53 and Ki-67 were tested in partial samples. The Ki-67 index of the surgical samples was <2%. However, partial OCVMs have been shown to exhibit positivity with CD99, CD34 and Vimentin.

### Follow-Up Results

All 35 patients completed the follow-up, which had a median period of 64.5 months. There were 35 operations: transnasal endoscopic approach in 22 cases (62.9%) and transfronto-orbital approach in 13 cases (37.1%). No patients experienced recurrence, and a total of 9 patients (25.7%) experienced postoperative complications. Vision improved postoperatively in 17 cases (48.6%), and there were no apparent visual changes after surgery in 9 cases (25.7%).

The surgical prognosis and clinical characteristics of Type I and Type II OCVMs are shown in **Table 1**.

**Table 1:** Clinical characteristics and surgical prognosis of type I and type II cavernous hemangioma.

INDEX	Type I (Group n=9)	Type II (Group n=26)	All patients (group N=35)
Sex			
Male	3 (33.3%)	7 (26.9%)	10 (28.6%)
female	6 (66.7%)	19 (73.1%)	25 (71.4%)
age (Y)	44.7 ± 6.6	43.0 ± 10.9	43.3 ± 10.3
first visit			
vision loss	9 (100.0%)	26 (100.0%)	35 (100.0%)
Exophthalmos	0 (0%)	0 (0%)	0 (0%)
Side			
left	3 (33.3%)	10 (37.9%)	13 (37.1%)
right	6 (66.7%)	16 (62.1%)	22 (62.9%)
Acute or subacute visual loss	8 (88.8%)	15 (57.7%)	23 (65.7%)
Fundus condition			
Edema of optic disc	4 (44.4%)	10 (38.5%)	14 (40.0%)
Atrophy of optic disc	5 (55.6%)	5 (19.2%)	10 (28.6%)
Mean diameter of tumor (cm)	0.77 ± 0.17	1.02 ± 0.24	0.95 ± 0.25
Missed diagnosis of imaging	8 (88.8%)	12 (46.2%)	20 (57.1%)
Misdiagnosed as optic neuritis	8 (88.8%)	12 (46.2%)	20 (57.1%)
Operation approach			
Craniotomy	1 (11.2%)	12 (46.2%)	13 (37.1%)
Transnasal endoscopic surgery	8 (88.8%)	14 (53.8%)	22 (62.9%)
Prognosis of operation			
Improved vision	4 (44.4%)	13 (50.0%)	17 (48.6%)
Decreased vision	2 (22.2%)	7 (26.9%)	9 (25.7%)
no change in vision	3 (33.3%)	6 (23.1%)	9 (25.7%)
surgical complications	3 (33.3%)	11(42.3%)	14 (40.0%)
Median follow-up time (M)	40.0	103.5	64.5
recrudescence	0 (0%)	0 (0%)	0 (0%)

### Typical Cases and Complication Analysis

**Figure 1:**
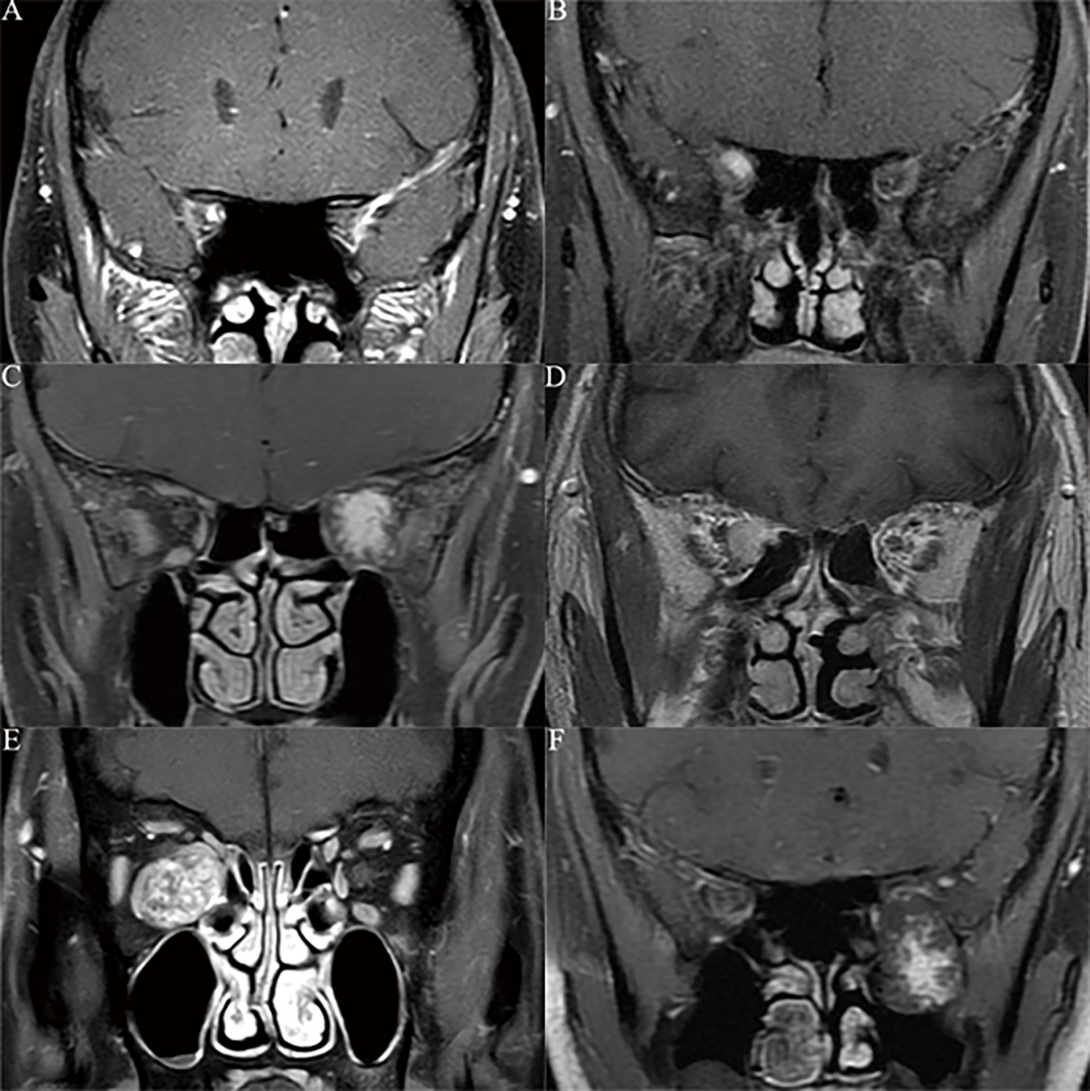
According to optic nerve MRI and intraoperative confirmation, we found the common location classification of orbital cavernous hemangioma. **(A)** MRI of optic nerve showed the tumor is located under the optic nerve sheath in the orbital apex area. **(B)** MRI of optic nerve showed the tumor is located in the common tendon ring of the orbital apex region. **(C)** MRI of optic nerve showed the tumor is located inside the muscle cone of the orbit. **(D)** MRI of optic nerve showed the tumor is located outside the muscle cone of the orbit. **(E)** MRI of optic nerve showed the tumor involved both the inside and outside communication region of the muscle cone. **(F)** MRI of optic nerve showed the tumor involved both the inside and outside communication region of the orbit. On all contrast-enhanced MRI images, we can see that cavernous hemangioma varies in size according to its location in the orbit, showing homogeneous or patchy enhancement.

**Figure 2:**
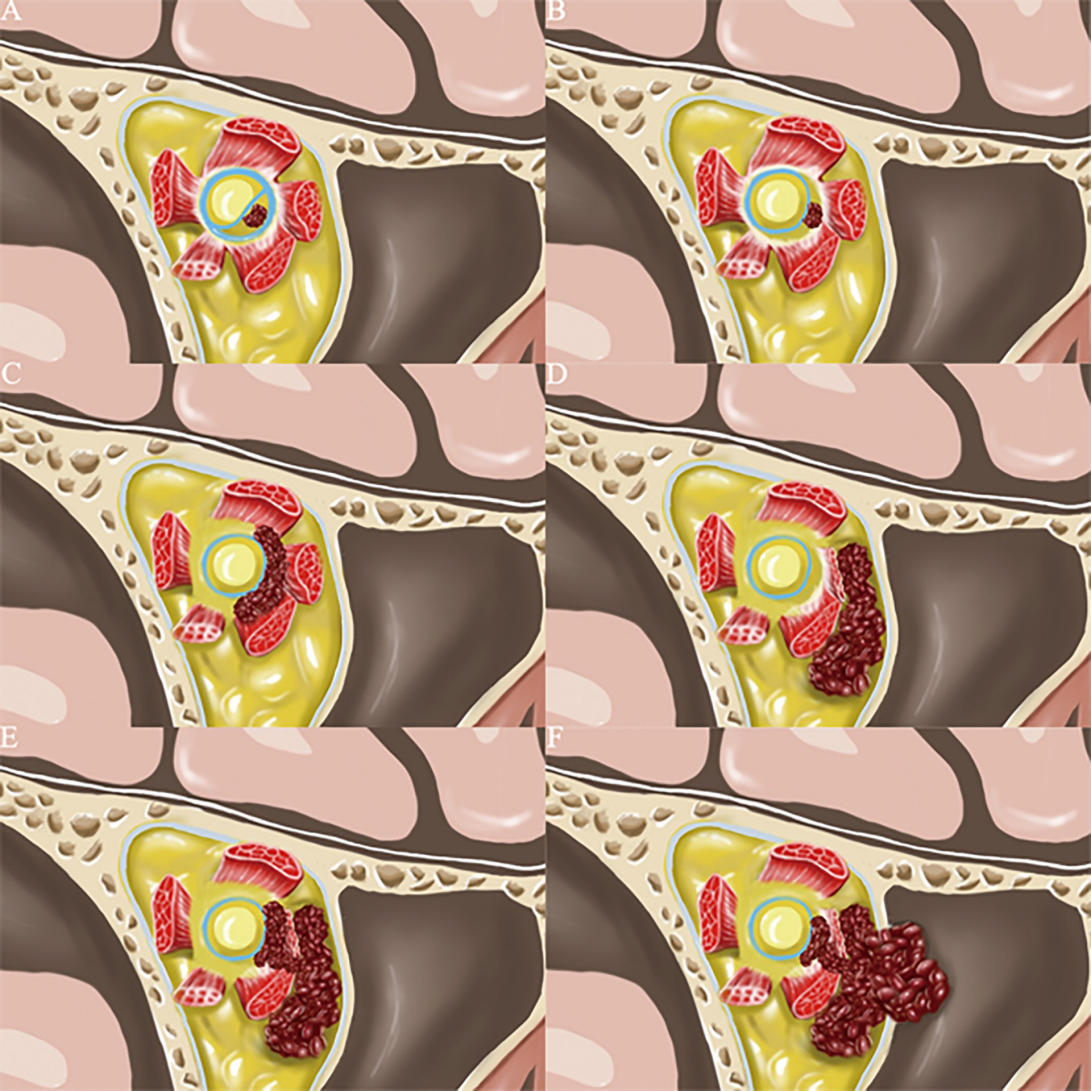
According to the location of cavernous hemangioma in the orbit, we have drawn the 6 most common clinical types. **(A)** (Type I): The tumor is located under the optic nerve sheath near the optic canal in the orbital apex area. **(B)** (Type II): The tumor is located in the common tendon ring of the orbital apex region and not under the optic nerve sheath. **(C)** (Type III): The tumor is located inside the muscle cone of the orbit. **(D)** (Type IV): The tumor is located outside the muscle cone of the orbit. **(E)** (Type V): The tumor involved both the inside and outside communication region of the muscle cone. **(F)** (Type VI) The tumor involved both the inside and outside communication region of the orbit, and there is a little chance of breaking through the orbital periosteum.

The typical OCVM locations were identified according to the optic nerve MRI and following intraoperative confirmation (**Figure 1**). Based on this information and our clinical experience, the OCVMs were classified into one of six types (I–VI) according to the orbital position of the tumour (**Figure 2**). Commonalities in the prognosis and clinical characteristics of surgery in Type I and Type II OCVMs were noted.

Type I: The OCVM is located under the optic nerve sheath of the orbital apex. The tumour has a complete envelope and generally does not penetrate the optic nerve sheath. The tumour is less than 0.77 ± 0.17 cm in diameter and compresses the optic nerve. It is easy to miss the diagnosis in MRI and CT of the brain.

**Figure 3:**
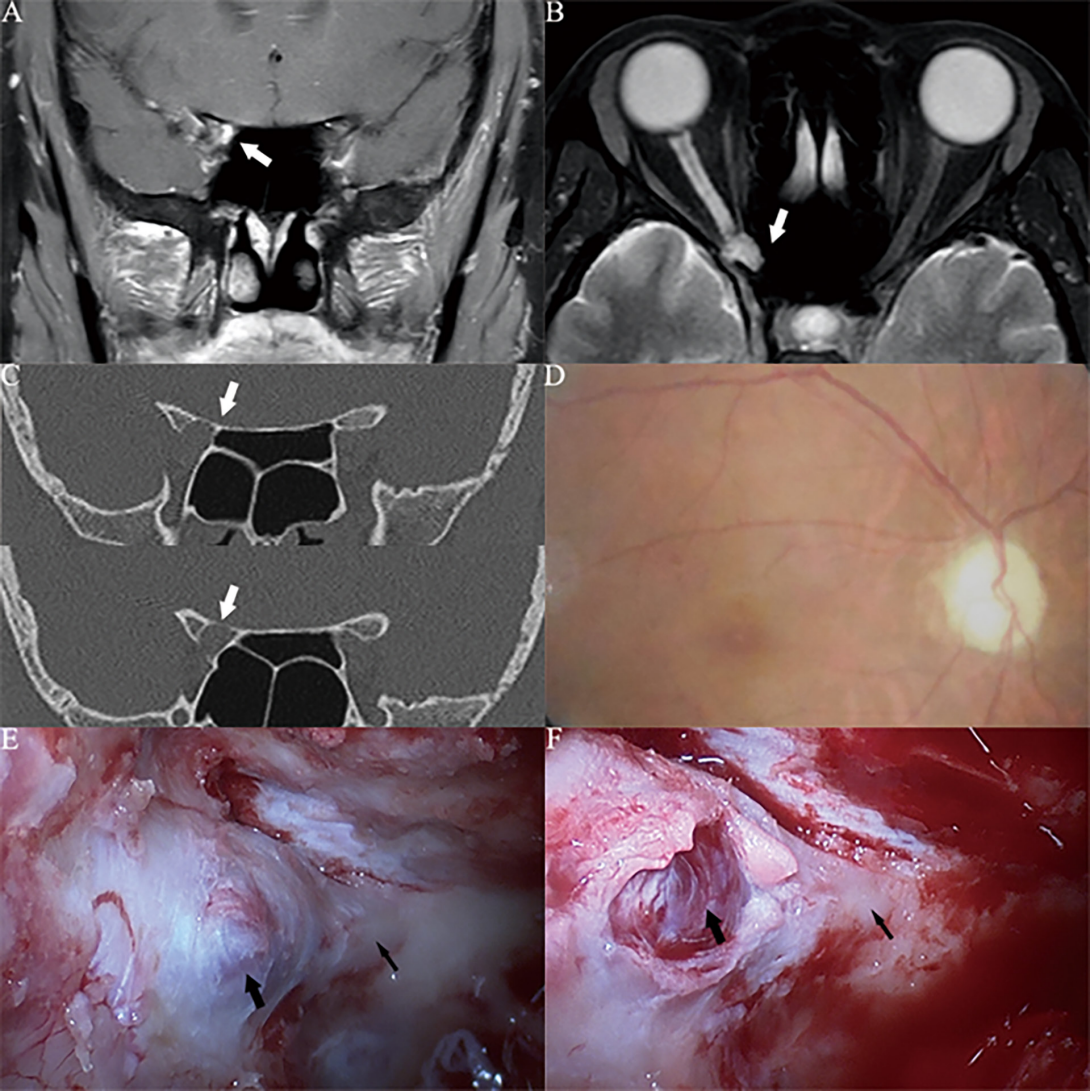
Case1. A 46-year-old female presented with her right eye vision loss for 2 months and sudden loss of right eye vision for 2 weeks. She was misdiagnosed as optic atrophy in the local hospital and was given hormone therapy. **(A, B)** Optic nerve MRI showed that the right optic nerve sheath occupies, uniformly enhanced, the tumor diameter is less than 1cm (white thick arrow). **(C)** CT of the optic canal shows that the right optic canal is wider than the left (white thick arrow). **(D)** Fundus photography shows atrophy of the right optic disc. **(E)** The right optic canal (black thin arrow) can be seen under the vision of nasal endoscopic surgery, and the space occupying under the optic nerve sheath near the orbital foramen is blue stained. **(F)** Incision of the optic nerve sheath reveals that a cavernous hemangioma is located under the optic nerve sheath (black thick arrow), behind which is the cranial foramen of the optic canal (black thin arrow).

**Figure 4:**
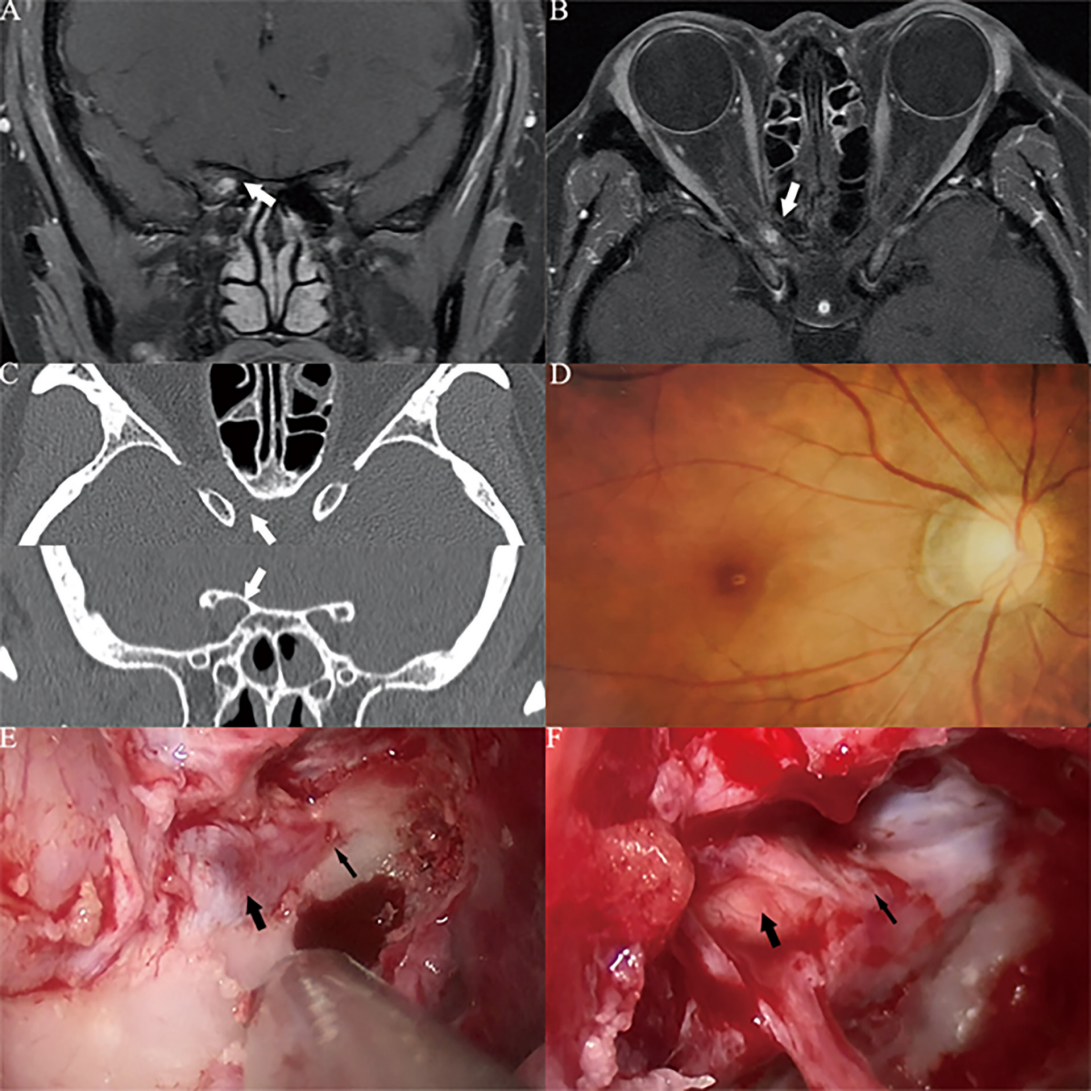
Case2. A 32-year-old female presented with her right eye vision loss for 8 months and sudden loss of right eye vision for 2 weeks. She was misdiagnosed as optic atrophy in the local hospital and was given hormone therapy. **(A, B)** Optic nerve MRI showed that the right optic nerve sheath occupies, uniformly enhanced, the tumor diameter is less than 1cm (white thick arrow). **(C)** CT of the optic canal shows that the right optic canal is wider than the left (white thick arrow). **(D)** Fundus photography shows atrophy of the right optic disc. **(E)** The right optic canal (black thin arrow) can be seen under the vision of nasal endoscopic surgery, and the space occupying under the optic nerve sheath near the orbital foramen is blue stained. **(F)** After resection of cavernous hemangioma, the right optic nerve was well protected (black thick arrow), and the cranial foramen of optic canal was behind it (black thin arrow).

**Figure 5:**
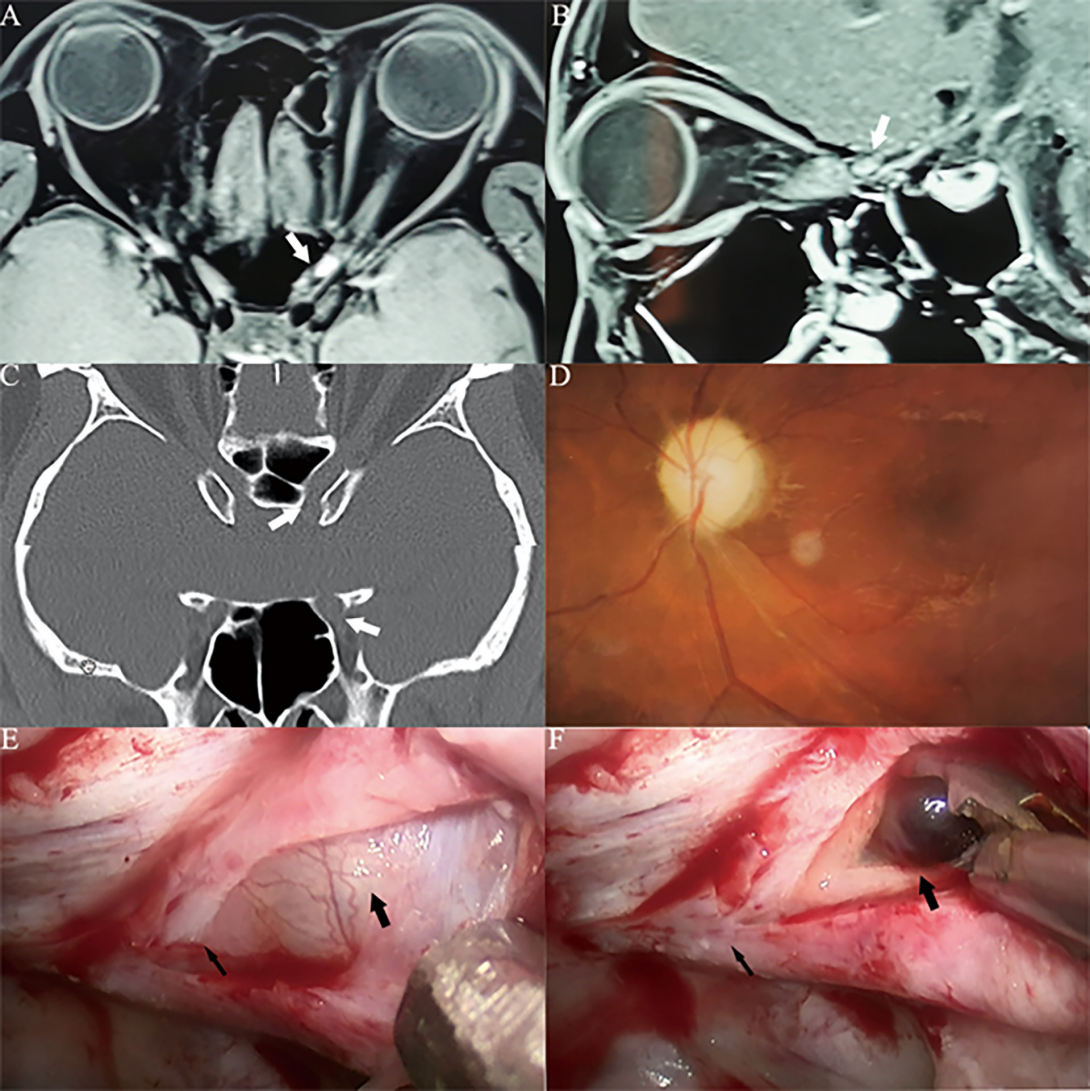
Case3. A 30-year-old male presented with his left eye vision loss for 4 months and sudden loss of left eye vision for 3 weeks. He was misdiagnosed as optic atrophy in the local hospital and was given hormone therapy. **(A, B)** Optic nerve MRI showed that the left optic nerve sheath occupies, uniformly enhanced, the tumor diameter is less than 1cm (white thick arrow). The optic nerve was squeezed to the lateral side by the tumor and disappeared. **(C)** CT of the optic canal shows that the left optic canal is wider than the right (white thick arrow). **(D)** Fundus photography shows atrophy of the right optic disc. **(E)** The left optic canal (black thin arrow) can be seen under the vision of nasal endoscopic surgery, and the space occupying under the optic nerve sheath near the orbital foramen is blue stained. **(F)** Incision of the optic nerve sheath reveals that a cavernous hemangioma is located under the optic nerve sheath (black thick arrow), behind which is the cranial foramen of the optic canal (black thin arrow), cavernous hemangioma is dark red, and a history of subacute bleeding is considered.

The OCVMs in cases 1, 2 and 3 (**Figures 3**–**5**) were all located under the optic nerve sheath in the orbital apex area. The tumours were very small in diameter and had been missed in imaging. Hormonal treatment had been given in local hospitals to treat acute or subacute neuropathy based on a diagnosis of optic neuritis. After hormonal therapy was ineffective, many patients experienced acute or subacute visual loss as a result of OCVM haemorrhage, which led to further investigation and diagnosis using optic nerve MRI. In case 3 (**Figure 5**), characteristic OCVM bleeding occurred; this is associated with a poor prognosis.

Type II: The OCVM is located in the area of the common tendon ring of the orbital apex and has a complete capsule. The tumour body is less than 1.02 ± 0.24 cm in diameter. The tumour is generally located in the muscle cone and does not extend through the common tendon ring and orbital periosteum. The total tendon ring contains extraocular muscles; the optic, oculomotor, trochlear, and abducent nerves; and other structures. The space in the entire tendon ring is narrow, and the tumour is small; it is therefore easy to miss the diagnosis in MRI and CT of the brain. However, once bleeding occurs, there will be acute or subacute visual loss and even the occurrence of orbital apex syndrome.

**Figure 6:**
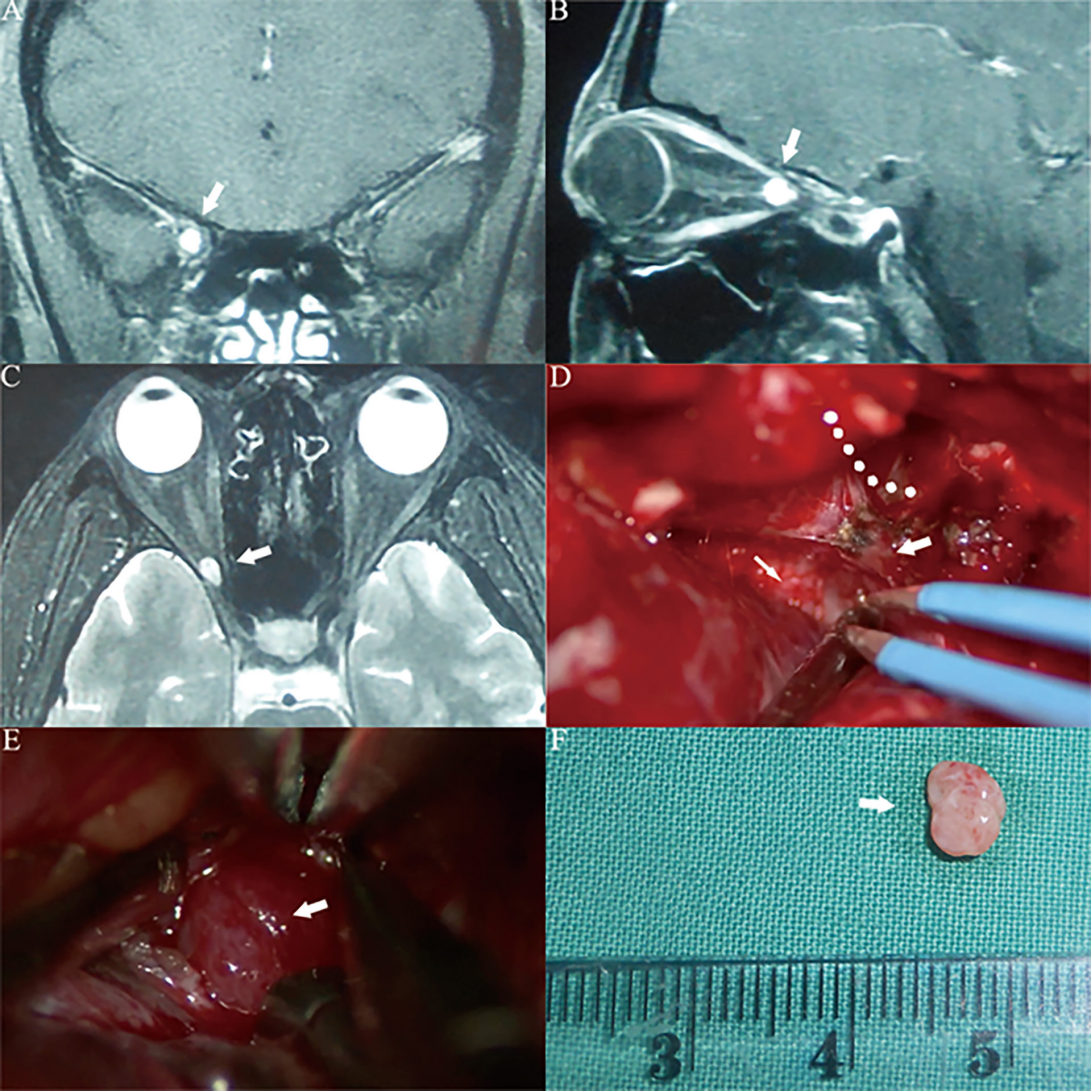
Case4. A 34-year-old female presented with her right eye vision loss for 2 months and sudden loss of right eye vision for 2 weeks. She was misdiagnosed as optic atrophy in the local hospital and was given hormone therapy. **(A–C)** MRI of the optic nerve showed a space-occupying lesion in the ring of the common tendon in the right orbital apex area, with uniform enhancement, and the tumor diameter was less than 1 cm (white thick arrow). **(D)** The tumor is located lateral the optic nerve. We used a right frontal-orbital approach craniotomy to remove the tumor. Abrasion of the optic canal bone shows the right optic nerve (white thin arrow), abrasion of the superior orbital fissure bone shows the right superior orbital fissure (white thick arrow), and the orbital periosteum at the orbital apex is incised lateral the superior orbital fissure along the dotted line. **(E)** It can be seen that the tumor capsule is intact, light red, and located lateral the right optic nerve (white thick arrow). **(F)** The tumor can be seen after complete resection. The actual tumor diameter is about 5mm (white thick arrow).

Missed diagnosis, misdiagnosis, insidious onset, and acute and subacute visual loss are characteristics of Type II OCVM. Due to the location of the OCVM in the common tendon ring, the extent of the operative field for transnasal endoscopic surgery is limited, and microscopic craniotomy is preferred. Case 4 (**Figure 6**) is an example of such an operation. Craniotomy with a transfrontal orbital approach is often used. Removing the brow bone and the frontal temporal bone can increase the operative field in the orbital apex; this can in turn reduce traction on the orbital apex structures.

**Figure 7:**
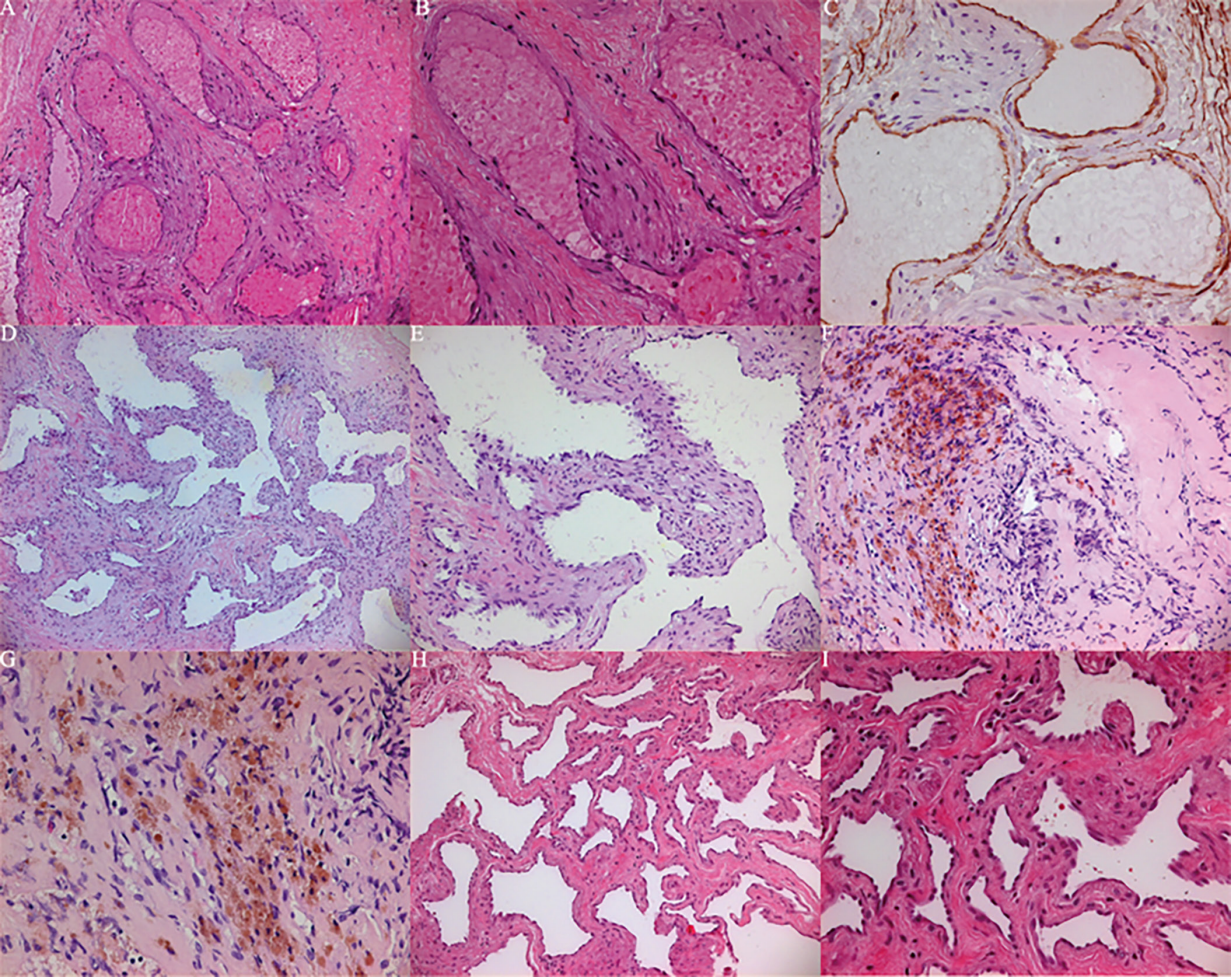
**(A–C)** Histopathological examination showed that case 1 proliferates and expands spongy blood vessels, and the cut surface is spongy. **(A)** (HE 100), **B** (HE 200), **(C)** (CD34+). **(D, E)** histopathological examination showed that case 2 proliferates and expands spongy blood vessels, and the cut surface is spongy. **(D)** (HE 50), **(E)** (HE 100). **(F, G)** Histopathological examination showed that case 3 proliferates and expands cavernous vessels accompanied by previous bleeding. **(F)** (HE 100), G (HE 200). **(H, I)** Histopathological examination showed that case 4 proliferates and expands spongy blood vessels, and the cut surface is spongy. **(H)** (HE 100), **(I)** (HE 200).

The histopathological examination of specimens in cases 1–4 showed proliferated and expanded spongy blood vessels, and the cut surface was porous. In Case 3, it proliferated and expanded cavernous vessels accompanied by previous bleeding. The OCVM pathology does not have any rare manifestations; all have the characteristics of OCVMs, and the previous bleeding in the pathological pictures can better explain the acute change in the disease (**Figure 7**). The Ki-67 index of surgical samples was <2%. However, partial OCVMs have been shown to exhibit positivity with CD99, CD34 and Vimentin (9).

Type III: The OCVM is located inside the muscle cone of the orbit and has a complete capsule. The OCVM is generally large and exerts a space-occupying effect; consequently, patients often have exophthalmos. As the tumour is commonly located in the muscular cone in a deep location, the operative field is narrow. Due to the relatively large size of the tumour and the clinical presentation of exophthalmos, the tumour is usually diagnosed *via* brain CT and MRI.

Type IV: The OCVM is located outside the muscle cone of the orbit. The tumour has a complete envelope and generally does not extend into the orbital periosteum. The OCVM is generally large and exerts a space-occupying effect; consequently, patients often have exophthalmos. The tumour is commonly located in a shallow position outside the muscle cone and rarely compresses the optic nerve; thus, vision is typically not affected. Due to the location of the tumour, more surgical approaches are available. The tumour is usually diagnosed *via* brain CT and MRI due to its relatively large size and the clinical presentation of exophthalmos.

Type V: The OCVM involves both the inside and outside communication region of the muscle cone. The tumour has a complete envelope and generally does not extend through the orbital periosteum. The OCVM is usually large and exerts a space-occupying effect; consequently, patients often have exophthalmos. The base of the tumour is commonly located deep in the muscular cone and often compresses the optic nerve, resulting in very poor vision.

Type VI: The OCVM involves both the inside and outside communication region of the orbit, and there is a slight chance of breaking through the orbital periosteum. The tumour body breaks through the orbital periosteum to the surrounding space. The entire tumour body can extend into the sphenoid sinus, ethmoid sinus, pterygopalatine fossa and even the cavernous sinus. Type III–VI OCVMs are generally large and exert a space-occupying effect; consequently, patients often have exophthalmos. Surgery requires a multidisciplinary approach involving various specialties.

## Discussion

OCVM is a benign vascular disease; it is a vascular malformation characterized by abnormal changes in the vascular cavity. In the past, some authors have classified it as a type of benign tumour. As the OCVM often occurs in the muscle cone behind the eyeball, it can cause axial exophthalmos and slowly grow to compress the optic nerve or eyeball, resulting in loss of vision. Therefore, early diagnosis and treatment of patients with OCVM are recommended as technology permits in order to avoid irreversible damage to visual function caused by advanced tumours. The current description of this orbital tumour’s location involves using the muscular cone as an anatomical boundary (divided into the inner and outer muscular cones) or using the optic nerve as a reference point to describe the relative position of the tumour (10).

However, there is no consensus on how to describe the different depths of the tumour within the orbit. Some authors provide an anatomical basis for the clinical, surgical approach, dividing the bony orbit into peripheral, posterior and orbital apexes: (1) the orbital apex is marked by the posterior ethmoid sinus; (2) the posterior part of the eyeball is marked by the anterior ethmoid sinus; and (3) the peripheral is the part of the orbit before the posterior part of the eyeball.

This method is based only on the anatomy of the bony orbit. However, during surgery, the surgeon needs to consider the bony anatomical landmarks of the orbit and the soft tissue landmarks, such as the eyeball and the extraocular muscles. If only the bony orbit is used as a partition, the specific relationship between each partition and the eyeball cannot be accurately represented. Therefore, each OCVM was classified as one of six types based on the orbital position of the tumour and its positional relationship to the bony orbit, the eyeball, the optic nerve canal within the orbit and the communication area inside and outside the orbit.

There have been many reports on the different surgical approaches for orbital tumours: the conjunctival approach to the orbital opening, the skin approach to the orbital opening, the lateral orbital opening, combined internal and external orbital openings and transnasal endoscopic approaches (11–16).

At present, the removal of OCVMs *via* nasal endoscopy is becoming increasingly accepted and promoted by the majority of doctors. Transnasal endoscopic surgery induces minimal trauma and leads to a faster patient recovery and limited surgical damage. However, for many OCVMs, limitations remain (17).

The current discussion on minimally invasive surgery for orbital tumours mainly focuses on the choice of surgical approach; that in turn mainly depends on the nature, size and location of the intra-orbital tumour (18–21).

Our department, which is led by neurosurgery and assisted by ophthalmology, has treated a total of 35 patients with OCVMs in clinical practice. All of these were patients in whom ophthalmic surgery alone was not indicated following ophthalmology outpatient screening. The chosen approach involved neurosurgery combined with ophthalmology to perform craniotomy and transnasal endoscopic surgery. Following a review of 17 years of data, including information on different surgical approaches and patient characteristics from the ophthalmologists, some interesting phenomena and common points were identified in certain cases.

The authors of this study believe that the clinical history and surgical prognosis of Type I and Type II patients have some commonalities: (1) in the majority of cases, the average tumour diameter was <1.0 cm (0.95 ± 0.25 cm; Type I: 0.77 ± 0.17 cm; Type II: 1.02 ± 0.24 cm); (2) as the tumour was small, it was often missed by ordinary cranial MRI and CT. The average missed diagnosis rate is 57.1% (Type I: 88.8%; Type II: 46.2%); (3) the patients showed no clinical signs of exophthalmos, with only visual loss in a single eye as a manifestation. All of the patients were first diagnosed with decreased vision (100%); (4) the majority of patients presented with optic disc atrophy (Type I: 55.6%; Type II: 19.2%) and optic disc edema (Type I: 44.4%; Type II: 38.5%) and were initially treated based on a diagnosis of optic neuritis (Type I: 88.8%; Type II: 46.2%), although the effect of treatment was limited. After a sudden drop in visual acuity, tumours were identified *via* orbital or optic nerve MRI in the outpatient clinic; (5) the tumours were small and deep. Surgery was technically challenging, often requiring neuronavigation techniques for successful tumour removal; and (6) transnasal endoscopy and craniotomy could be used for surgical removal. However, the probability of postoperative recovery of vision was small, the probability of complications and further visual loss was high, and the risk of orbital apex syndrome after surgery was high.

The authors of this study propose that this kind of OCVM often occurs in the optic nerve sheath at the orbital apex or is confined to the muscle cone of the common tendon ring. Space is limited at the anatomical location of these tumours, restricting tumour growth, and the average diameter of the tumour at this location is <1.0 cm.

Although the space-occupying effect of the OCVM is not great, tumours at this location usually involve the optic nerve. In most patients, visual acuity decreases slowly and is accompanied by optic disc edema, potentially contributing to a misdiagnosis of optic neuritis in the outpatient setting. However, when an OCVM in this location bleeds due to the limited space in the tumour’s location, the clinical symptoms and signs will change dramatically, resulting in severe damage to the optic nerve and marked visual loss for the patient.

When this acute change in visual acuity occurs, the outpatient doctor may carry out an orbital or optic nerve MRI resulting in the correct diagnosis. The history of OCVM is not accurately reported in the relevant literature. Still, it is known that bleeding is the major complication of this type of tumour, and the probability of rebleeding is high.

Ren Jian (22) reported that the annual bleeding rate in paediatric patients within the nervous system was 8.2%/patient/year. After an initial clinically significant bleeding event, the annual rebleeding rate increased to 30.7%/patient/year (2.8% and 7.4% in adult patients, respectively).

Depending on the location of the OCVMs, craniotomy and transnasal endoscopic surgery are performed in patients with Type I and Type II OCVMs. When the tumour is located on the medial or inferior side of the optic nerve, the medial side of the common tendon ring and close to the orbital periosteum, a transnasal endoscopic surgical approach is adopted. Under the endoscope, only the optic nerve canal and the bones of the orbital apex need to be removed.

It is evident that blue staining occurs in the dura of the optic nerve sheath. After cutting the sheath at the tumour, the optic nerve is protected while the tumour is carefully separated.

When the tumour is located in the superior or lateral side of the optic nerve, or the depth of the common tendon ring is in position, craniotomy with the transfrontal orbital approach can open the optic nerve sheath or the common tendon ring to remove the tumour. Craniotomy completely exposes the superior orbital fissure and optic nerve, removes the bones of the optic canal and superior orbital fissure and completely exposes the structures of the orbital apex area. It is imperative to cut the common tendon ring structure. The orbital periosteum of the orbital apex is often cut using a lateral incision at the superior fissure.

Note that the trochlear nerve usually crosses over the ring of the tendon. To avoid postoperative orbital apex syndrome, the orbital apex structures (including the extraocular muscles and the optic, oculomotor, trochlear and abducent nerves) must be protected during surgery.

Most cases of missed diagnosis or misdiagnosis occur in the ophthalmology or neurology departments of the primary hospital; thus, the question of how to effectively screen at first presentation is worth considering. In the incidence of a decrease in monocular vision, combined with a lack of response to hormonal therapy for presumed optic neuritis in the clinical setting, optic nerve MRI is an effective investigation for identifying diseases such as OCVM. Once OCVM haemorrhage has occurred, visual acuity will be irreversibly damaged.

In the pathological pictures of some typical cases, there will be characteristic manifestations of tumour haemorrhage. OCVM is a type of venous malformation in the strictest sense, although it is a vascular malformation associated with low flow. Despite our experience in OCVM surgery, it remains unclear why Type I OCVM has a higher bleeding rate than Type II OCVM or even other OCVM types.

The medical history of OCVM has not been adequately studied, and tumour characteristics at each site are not the same. In addition, the overall sample in the present study is relatively small.

Ren Jian (23) reported that patients with cavernous haemangioma (CH) in the spinal canal also have CH of the cranium. However, none of the 35 patients included in the present study had combined CH of the cranium. Some authors report that intra-OCH will have evident bone destruction apparent on CT (24). In the present research, bone proliferation, destruction and resorption were rarely seen. In addition, some authors have proposed that the complications of OCVM surgery are low (25); however, in our experience, the incidence of surgical complications was relatively high.

## Conclusion

Patients with Type I and Type II OCVMs, which have an insidious onset, are the most complex cases. They are associated with a high rate of misdiagnosis and missed diagnosis.

Acute and subacute reduction in visual acuity are usually caused by tumour haemorrhage. The difficulty of the operation and poor prognosis of postoperative vision are the characteristics of this tumour. Based on the present study, transnasal surgery can be used to remove OCVMs. Nevertheless, treatment complications of missed diagnosis and misdiagnosis are hard to avoid, and the incidence of surgical complications remains high.

## Data Availability Statement

The original contributions presented in the study are included in the article/supplementary material. Further inquiries can be directed to the corresponding author.

## Ethics Statement

The studies involving human participants were reviewed and approved by Tongren Hospital of China Capital Medical University. The patients/participants provided their written informed consent to participate in this study.

## Author Contributions

PY have made substantial contributions to the conception or design of the work. H-CL and YL acquisition, analysis, or interpretation of data for the work. PY, J-LZ, and JR have been involved in drafting the work or revising it critically for important intellectual content. L-BJ and H-GL have given final approval of the version to be published. EQ and JK agree to be accountable for all aspects of the work in ensuring that questions related to the accuracy of integrity of any part of the work are appropriately investigated and resolved. All authors contributed to the article and approved the submitted version.

## Conflict of Interest

The authors declare that the research was conducted in the absence of any commercial or financial relationships that could be construed as a potential conflict of interest.

The handling Editor, JZ, has declared a shared parent affiliation with the authors at the time of review.

## Publisher’s Note

All claims expressed in this article are solely those of the authors and do not necessarily represent those of their affiliated organizations, or those of the publisher, the editors and the reviewers. Any product that may be evaluated in this article, or claim that may be made by its manufacturer, is not guaranteed or endorsed by the publisher.
